# A Modified Preserved Nasal and Lacrimal Flap Technique in Endoscopic Dacryocystorhinostomy

**DOI:** 10.1038/s41598-017-07364-9

**Published:** 2017-07-28

**Authors:** Wenyan Peng, Bowei Tan, Yandong Wang, Haiying Wang, Zhonghao Wang, Xuanwei Liang

**Affiliations:** 1 State Key Laboratory of Ophthalmology, Zhongshan Ophthalmic Center, Sun Yat-sen University, Guangzhou, 510060 P.R. China; 2 Brookdale University Hospital and Medical Center, Brooklyn, NY 11212 USA

## Abstract

Here we describe a modified preserved nasal and lacrimal mucosal flap technique in endonasal endoscopic dacryocystorhinostomy (EES-DCR) for patients with epiphora secondary to primary acquired nasolacrimal duct obstruction (PANDO) and evaluate its outcomes. Twenty-five patients with PANDO were retrospectively reviewed. Modified preserved nasal and lacrimal mucosal flap technique in EES-DCR was applied in all 27 eyes of 25 patients. The patients were evaluated with objective (anatomical patency) and subjective (symptomatic cure) success rates within the duration of follow-up. In the present study, all of the patients’ surgical procedures were successful. There were 2 cases of flap dislocation from the rhinostomy site 1 week post-operation. After a mean follow-up of 4.9 ± 1.8 months, the success rate of anatomical patency was 100% (27/27) and the success rate of symptomatic cure was 92.6% (25/27). No significant complications occurred intraoperatively. We concluded that the modified preserved nasal and lacrimal mucosal flap technique in EES-DCR for treating PANDO is simple and safe, can effectively cover the bare bone around the opened sac, and provide a similar or even better clinical outcome compared with other routine treatment techniques used for this condition.

## Introduction

Patients with primary acquired nasolacrimal duct obstruction (PANDO) may develop persistent epiphora, chronic or acute dacryocystitis, conjunctivitis or chronic conjunctival injection, which often require surgical treatment. To treat PANDO, dacryocystorhinostomy (DCR) is a surgical method used to re-establish tear flow from the lacrimal system to the nasal cavity, which may be applied either intranasally or externally. However, due to the instrumentation and technical difficulties in visualizing the surgical site and achieving effective mucosal tissue and bone removal, the popularity of intranasal DCR was limited. Lacrimal bypass surgery was performed almost exclusively through an external incision as described by Toti, and then became the “gold standard” technique to treat PANDO for almost the last 100-years^1^. With the development of the rigid fiberoptic endoscope and application of appropriate instrumentation, the first modern endonasal endoscopic DCR procedure was described by McDonogh and Meiring in 1989^2^. Since then, endonasal endoscopic DCR (EES- DCR) was performed more frequently in PANDO and successful results were reported.

Even though different methods to perform endonasal endoscopic surgery have been reported from 1990 to 1997, the success rate of the endonasal approach was unsatisfactory and not comparable with that of the external approach^3^. The most common cause of surgical failure in endonasal endoscopic DCR is that the neo-ostium is obstructed by either granulation tissue or synechia formed postoperatively^4, 5^.

How to create a large bony ostium and minimize postoperative scarring, stenosis, and maintain sustained patency of the ostium is of key importance in performing EES-DCR. Therefore, numerous new methods have been invented to increase the surgical success rate of EES-DCR. These new techniques, including various lasers, surgical electrodes, powered drills, or punches were applied to remove the bone covering the lacrimal sac and duct^6–10^; mostly nasal mucosal flaps were preserved after wide resection to decrease the formation of granulation tissue and synechia. By fashioning a U-shaped flap over the ostia meatus, which purportedly led to primary healing without granulation, Wormald achieved more than 95% surgical patency rate for an average of 11 months^11^. However, the techniques of U-shaped and L-shaped nasal mucosal flaps sacrificed most of the central part of the flap^12, 13^ and it hardly covered the widely exposed bone. Furthermore, the small portion of nasal mucosal flap was easily torn or even lost.

Herein, we report a modified technique to create nasal and lacrimal mucosal flaps in EES-DCR procedure. This modification simplifies the technical challenges, preserves the entire nasal and lacrimal mucosal flap, maximizes coverage of the bare bone and avoids the flap mobility and loss.

## Participants and Methods

### Ethics Statement

This study was approved by the institutional review board (IRB) of ZhongShan Ophthalmic Center, Sun Yat-sen University, Guangzhou, China. And the methods were implemented in accordance with the approved guidelines. Informed consents were obtained from all patients.

### Participants

From June 2013 to December 2014, 27 EES-DCRs were performed at the Zhongshan Ophthalmic center, Sun Yat-sen University of China. Data were collected from 25 patients (8 male, 17 female, 10 right eyes, 13 left eyes, 2 bilateral cases). The average patient age was 40.6 years (range 6–70 years). Every patient received a complete external and nasolacrimal system examination prior to operation, including dacryocystography and lacrimal system irrigation by the same ophthalmologist. Dacryocystography can directly visualize lacrimal sac atrophy, occupying lesions and/or foreign-body in the lacrimal drainage system and accurately localize the obstruction preoperatively in patients with nasolacrimal duct obstruction. We excluded patients with a previous lacrimal surgery history, a history of physical scars, lower eyelid malposition including ectropion or entropion, suspicion of malignancy, post-traumatic bony deformity, and previous facial fractures or nasal diseases, such as polyps and chronic sinusitis.

### Surgical technique of EES -DCR

**Figure 1:**
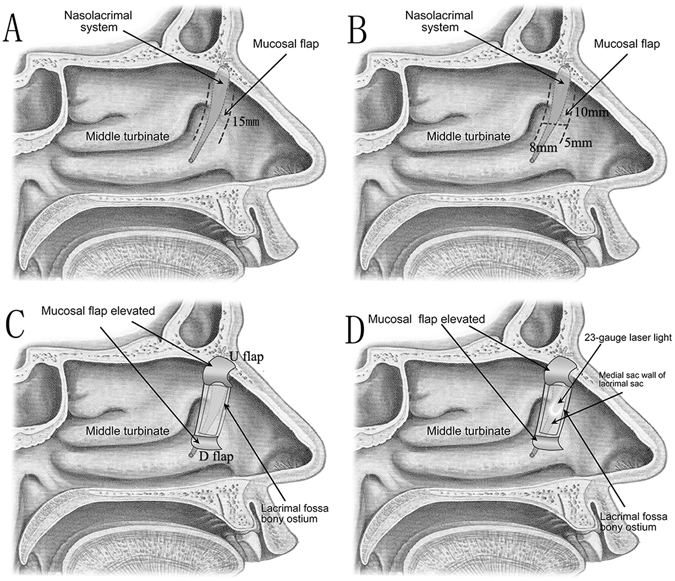
A Modified Preserved nasal and lacrimal mucosal flap Technique in Endoscopic Dacryocystorhinostomy. (**A** and **B**) Localization of nasal mucosal flap. (**C**) Open bony ostium, and expose lacrimal sac. (**D**) The 23-gauge laser light trans-illuminates and tents up the medial sac wall to confirm the surgical position in the lacrimal wall.

All procedures were performed under general anesthesia by the same surgeon (Dr. Xuanwei Liang). To provide sufficient topical decongestion and vasoconstriction, the nose was packed with cotton pledgets soaked in 4‰ adrenaline, followed by submucosal injection of mepivacaine 20 mg/ml and adrenaline 1:200,000 over the preconceived rhinostomy site^12^. Endoscopy was then performed with a 0° rigid endoscope (Stryker Surgical, Kalamazoo, MI, U.S.A.). The root of the middle turbinate and uncinate process was identified as a surgical landmark. A curvilinear incision was started 6 mm above the insertion of the middle turbinate, and then ceased at the top of inferior turbinate insertion, locating anteriorly to the uncinate process (Fig. 1A). A parallel curvilinear incision was made about 8 mm distal from the first incision, and then a nearly 15 mm length narrow strip mucosal flap was formed and elevated with a freer elevator. The mucosal flap was about 8 mm (width) ×15 mm (length) (Fig. 1A). Next, the strip mucosal flap was cut in the middle, creating such an“H- shape” flap: the upper flap (U flap) was about 10 mm long, and the lower flap (D flap) was about 5 mm long (Fig. 1B). Then, the U and D flap were folded up and down respectively to expose lacrimal fossa bone, which was subsequently removed by a bone-biting forceps or drill and a 12 mm × 10 mm bony ostium was created (Fig. 1C). After the medial sac wall of the lacrimal sac was exposed, instead of using a bowman probe which may form a false passage occasionally, we utilized a 23-gauge laser light pipe to pass through either the superior or inferior canaliculus into the lacrimal sac and trans-illuminate the lacrimal sac wall, and tent up the medial sac wall so that we could confirm the surgical position in the lacrimal wall (Fig. 1D).

**Figure 2:**
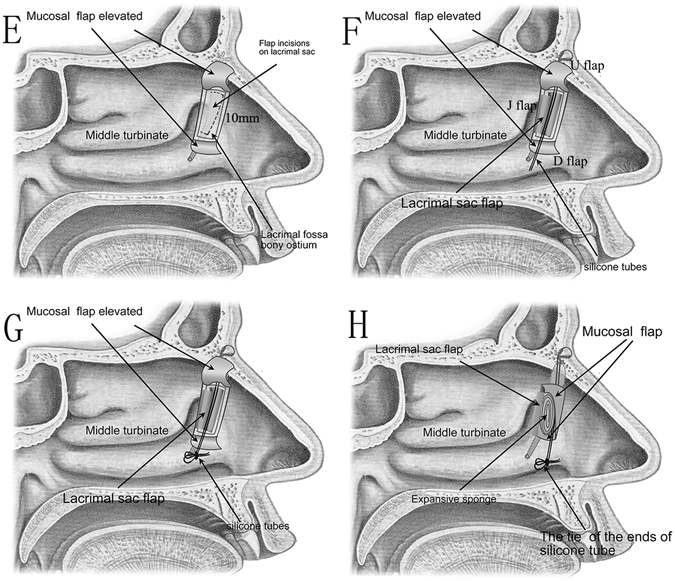
A Modified Preserved nasal and lacrimal mucosal flap Technique in Endoscopic Dacryocystorhinostomy. (**E**) Localization of the lacrimal sac flap incisions. (**F** and **G**) Lift up lacrimal sac flap, Silicone tubes are placed through lacrimal punctum after lacrimal flap is everted. (**H**) Mucosal flaps cover posterior, superior, anterior and inferior edge of the exposed bony ostium surface. Note the position of the expansive sponge, to maintain a patent ostium.

After the whole medial lacrimal sac mucosa was fully exposed, it was incised from its most superior position then straight down, ending at the level of the sac-duct junction. The incision was about 10 mm long. We then made two horizontal relaxing incisions at both ends to form a door “]” shape sac mucosal flap which was intended as the posterior edge of the bony ostium (J flap) (Fig. 2E). A silicone tube was placed from either the upper or lower punctum down in the nasolacrimal duct, and grasped with a Blakesley Forcep (Fig. 2F). The ends of the tubing were then tied and trimmed within the nasal cavity, and formed a continuous loop around the canaliculi (Fig. 2G). At the end step, we released all the flaps to cover the bony ostium without any tension, firstly the J flap covered the posterior edge of ostium, then the U flap covered the superior and anterior edge of ostium and finally the D flap covered the inferior edge of ostium. A suitable expansive sponge was placed at the osteotomy site and three mucosal flaps were compressively fixed at the conclusion of surgery (Fig. 2H).

### Postoperative Care

All subjects were treated with topical antibiotic-steroid drops and steroid nasal spray for 4 weeks after surgery. Patients were evaluated post-operatively at 1 week, 1 month, and every 3 months thereafter. All patients were instructed to have nasal spray with normal saline twice daily and lacrimal irrigation once daily to prevent crust formation upon discharge. The expansive sponge was removed from the operative site 1 week after surgery and the silicone tube was usually removed 4 to 6 weeks after surgery. Patency of the lacrimal drainage system was verified by endoscopic observation of fluorescein dye flowing from the eye through the surgical ostium into the nose in every follow up visit.

## Results

All 25 patients who underwent EES-DCR were reviewed. Two patients had procedures in both eyes so a total of 27 surgeries were performed, including 12 right eyes and 15 left eyes. The whole procedure took about 15–20 minutes, depending on the thickness of the lacrimal fossa bone that needed to be removed. No significant intraoperative complications were encountered. Two cases were found to have a U-flap dislocation from the original rhinostomy site one week after surgery. Both of them immediately had mucosal flap relocation under endoscopy with topical anesthesia and were packed with inflated sponges in outpatient clinic. After a mean follow-up of 4.9 ± 1.8 months, the success rate of anatomical patency was 100% (27/27). No sump syndrome caused by residual lacrimal sac was identified in any of our patients. Most of our subjects were symptom free after the procedure with only two patients complaining of epiphora on cold or windy days; the success rate of subjective cure was 92.6% (25/27). All the patients are still being followed, and most patients now undergo examination every 12-months with no further late failures reported.

## Discussion

Previous evidence showed endoscopic approaches were superior over conventional approaches in numerous aspects, such as avoidance of skin trauma, scar tissue formation, preservation of the orbicularis muscle pump function, improved hemostasis, decreased incidence of intraoperative bleeding and better visualization of anatomical structures, etc. Interest in EES-DCR has become increasingly popular since the 1990s. The success rate of EES-DCR reported in the literature ranges from 79.4% to 96%^10, 14–17^. The incidence of complications in endonasal DCR is not high, but has been reported: including infection, restenosis, hemorrhage, canalicular erosion, the blockage of the neo-ostium by granulation tissue or synechia and lacrimal sump syndrome^4, 18–20^. In order to increase the surgical success rate of endoscopic endonasal DCR, many new techniques have been applied in the last 20 years. The focus of these innovative approaches is how to preserve nasal mucosal flaps and create a large bony ostium^21–24^. Durvasula and Gatland reported that the formation of granulation tissue may be caused by the bare bone^25^. Kansu conducted a comparison study of surgical outcomes in EES-DCR with or without mucosal flaps and the results showed that the closure of bare bone with a nasal mucosal flap and an anastomosis between the lacrimal sac mucosa and the nasal mucosa decreased the formation of granulation tissue^3^. Mahendran introduced another option, like using a free mucosal flap to cover the bare bone in patients undergoing EES-DCR^26^. However, it also had limitations and/or disadvantages, such as being time-consuming, flap mobility and difficulty with survival of the free flap on the bare bone; especially when the created flaps cannot reach a full coverage of the bare bone in the ostium.

Therefore, we designed a modified technique in DCR in order to decrease the formation of granulation tissue and synechia, lessen the risk for subsequent scar or closure of the ostium, shorten surgical time and improve the long-term outcome. In our study, EES-DCR showed an anatomic success rate of 100% (27/27) after the primary surgery, with an open pathway evaluation by irrigation of the lacrimal system and nasal endoscopy. No residual lacrimal sac in any of the patients was identified to cause sump syndrome in our study. The rate of subjective success was 92.6% (25/27). Only 2 cases had epiphora, and only on cold or windy days. This may also be attributed to their concurrent xerophthalmia.

We designed the strip flap and divided it into two parts. The upper flap (U-flap) was about 10mm long and the lower flap (D-flap) was about 5mm long. The lacrimal sac flap covered the posterior edge of the ostium; the U-flap and D-flap covered the upper and lower edges of the ostium. This design can avoid vastly formation of lacrimal sump syndrome which could lead to DCR failure. We believe the preservation of the nasal mucosa and fashioning of a nasal “H-shaped” flap, as well as the designation of upper and lower flaps, enables primary healing along the posterior, superior, and inferior edges of the lacrimal sac and nasal mucosa, thus marsupializing the lacrimal sac into the lateral nasal wall. The complete coverage of the edges of the rhinostomy site by flaps is intended to minimize granulation tissue and synechia formation and prevent the occurrence of restenosis of the ostium, which is the most common cause of failure in endonasal DCR.

Whether a silicone stent should be applied intraoperatively is still controversial. In this study, a silicone stent is thought to help maintain the patency of the ostium during the packing process and to allow tear draining when irrigated. Some authors believe that silicone tube is too small in diameter to prevent future stenosis, and actually promotes granulation and therefore increases the failure rate^27^. However, some previous studies also showed that epithelial anastomosis and continuous fluid flow were necessary for maintaining a patent surgical rhinostomy, and both of these required silicone stent placement^28^. In our study, the silicone stents would be removed in the early stage (4 to 6 weeks after surgery) in order to minimize the risk of ostium closure by causing a granulomatous reaction^29, 30^.

The result of this study suggests that this nasal and lacrimal mucosal apposition allows the creation of a stable lacrimal ostium with healing by primary intention with minimal granulation tissue formation. This promotes a stable ostium with little shrinkage from 4 weeks to 12 months^31^. In this study, follow-up was limited to 6 months due to the anticipated drop-off in follow-up, and also because the majority of failures have been shown to occur early after surgery.

Generally, the time of epithelization of the bony ostium positively correlated with the extent of bare bone. With the bare bone maximally covered by nasal and lacrimal mucosal flaps in this technique, we can have more prompt epithelization of the bony ostium and achieve primary healing within a shorter period. However, this effective clinical outcome requires further study and a larger number of patients.

In the present study, we conclude that this modified technique in endonasal DCR may be recommended as a surgical procedure that achieves satisfactory objective and subjective success rates.
